# Folic acid deficiency increases sensitivity to DNA damage by glucose and methylglyoxal

**DOI:** 10.1093/mutage/geac003

**Published:** 2022-01-25

**Authors:** Leigh Donnellan, Bradley S Simpson, Varinderpal S Dhillon, Maurizio Costabile, Michael Fenech, Permal Deo

**Affiliations:** University of South Australia, Clinical and Health Sciences, Health and Biomedical Innovation, Adelaide, SA 5000, Australia; University of South Australia, Clinical and Health Sciences, Health and Biomedical Innovation, Adelaide, SA 5000, Australia; University of South Australia, Clinical and Health Sciences, Health and Biomedical Innovation, Adelaide, SA 5000, Australia; University of South Australia, Clinical and Health Sciences, Health and Biomedical Innovation, Adelaide, SA 5000, Australia; University of South Australia, Centre for Cancer Biology and SA Pathology, Frome Road, Adelaide, SA 5000, Australia; University of South Australia, Clinical and Health Sciences, Health and Biomedical Innovation, Adelaide, SA 5000, Australia; Genome Health Foundation, North Brighton, SA 5048, Australia; University of South Australia, Clinical and Health Sciences, Health and Biomedical Innovation, Adelaide, SA 5000, Australia

**Keywords:** nutrient interactions, micronuclei, glucose, folic acid, dicarbonyl stress, oxidative stress

## Abstract

Type 2 diabetes (T2D) is associated with elevated frequencies of micronuclei (MNi) and other DNA damage biomarkers. Interestingly, individuals with T2D are more likely to be deficient in micronutrients (folic acid, pyridoxal-phosphate, cobalamin) that play key roles in one-carbon metabolism and maintaining genomic integrity. Furthermore, it has recently been shown that deficiencies in these nutrients, in particular folic acid leaves cells susceptible to glucose-induced DNA damage. Therefore, we sought to investigate if the B lymphoblastoid WIL2-NS cell line cultured under folic acid-deficient conditions was more sensitive to DNA damage induced by glucose, or the reactive glycolytic byproduct methylglyoxal (MGO) and subsequent advanced glycation endproduct formation. Here, we show that only WIL2-NS cultured under folic acid-deficient conditions (23 nmol/l) experience an increase in MNi frequency when exposed to high concentrations of glucose (45 mmol/l) or MGO (100 µmol/l). Furthermore, we showed aminoguanidine, a well-validated MGO and free radical scavenger was able to prevent further MNi formation in folic acid-deficient cells exposed to high glucose, which may be due to a reduction in MGO-induced oxidative stress. Interestingly, we also observed an increase in MGO and other dicarbonyl stress biomarkers in folic acid-deficient cells, irrespective of glucose concentrations. Overall, our evidence shows that folic acid-deficient WIL2-NS cells are more susceptible to glucose and/or MGO-induced MNi formation. These results suggest that individuals with T2D experiencing hyperglycemia and folic acid deficiency may be at higher risk of chromosomal instability.

## Introduction

Micronutrients play a key role in maintaining genomic integrity because of their function as substrates or cofactors for enzymes involved in DNA synthesis and repair and DNA methylation [1]. The B group vitamins folate (B9), pyridoxine (B6), and cobalamin (B12), in their various bioactive forms such as methyl-tetrahydrofolate, pyridoxal-phosphate, and methyl-cobalamin, respectively, are critical for one-carbon metabolism. This process is required for the synthesis of deoxythymidine monophosphate from deoxyuridine monophosphate and remethylation of homocysteine to methionine and ultimately *S*-adenosyl methionine required for maintenance of DNA methylation patterns [2]. Deficiency in these vitamins is associated with genomic instability, caused primarily by uracil misincorporation into DNA and induction of micronuclei (MNi), which can be measured by the cytokinesis block micronucleus cytome (CBMNcyt) assay [1, 3, 4]. Moreover, deficiencies in pyridoxine and folic acid are associated with whole chromosome loss events, chromosomal rearrangement, and telomere attrition [4, 5]. Interestingly, deficiencies in these vitamins are associated with increased cellular susceptibility to DNA damage by other genotoxins. For example, B lymphoblastoid WIL2-NS cells grown in folic acid-deficient RPMI are more sensitive to radiation-induced DNA damage (1.5 Gy), experiencing a ~2-fold increase in MNi and nucleoplasmic bridges (NPBs) compared with controls grown in standard RPMI [6]. Therefore, it is important to consider how vitamin deficiencies may alter cellular responses to other genotoxic stresses, as various pathological conditions are associated with altered metabolic states.

Chromosomal instability (CIN) has been observed in individuals with type 2 diabetes (T2D). The CBMNcyt assay has been used to demonstrate that peripheral blood lymphocytes and buccal cells from T2D patients contain elevated levels of MNi and other DNA damage biomarkers [7–11]. Two recent meta-analyses have shown the meta-MR (mean ratio) for micronucleus frequency in lymphocytes in those with diabetes mellitus is 1.99 (*P* < .01; T2D) and 1.74 (*P* < .001; T2D and T1D) when compared with control groups [11, 12]. Despite this association, very few studies have investigated the potential underlying mechanisms. Interestingly, several studies have shown that individuals with T2D are more likely to be folate deficient than healthy controls, which may contribute to this observed CIN [13, 14]. We have recently shown that the reactive dicarbonyl compound methylglyoxal (MGO), which is elevated in T2D, increases MNi frequency in WIL2-NS and cultured primary peripheral blood lymphocyte by inducing chromosome malsegregation during mitosis [15]. MGO is formed as a byproduct of glycolysis, which may suggest elevated glycolytic flux, as seen in hyperglycemic states, may impact CIN in T2D [16]. High concentrations of glucose have also been shown to induce MNi and other chromosomal aberrations in folic acid deficient, normal human colon mucosal cell lines (NCM460, CCD841) and human liver (L02) cells [17]. Moreover, pyridoxal-phosphate-deficient HeLa cells and brain cells isolated from *Drosophila melanogaster* have also been shown to be sensitive to glucose-induced DNA damage [18, 19]. The cause for the interaction between glucose and folic acid was not investigated. However, the glucose-induced DNA damage in pyridoxal-phosphate-deficient HeLa cells was shown to be associated with the formation of advanced glycation endproducts (AGEs), a class of post-translational modifications, several of which are formed by MGO. Moreover, pyridoxal-phosphate acts a cofactor for the enzyme serine hydroxymethyltransferase, which is directly involved in folate mediated one-carbon metabolism (specifically the synthesis of 5,10-methylene tetrahydrofolate required for the synthesis of deoxythymidine monophosphate from deoxyuridine monophosphate). Therefore, alterations in this pathway may leave cells susceptible to DNA damage caused by elevated glucose and/or glycolytic byproducts which may adduct onto DNA or proteins involved in DNA synthesis/repair processes or mitosis [20–22].

The aim of this study was to determine if folic acid deficiency alters cellular sensitivity of WIL2-NS to glucose-induced DNA damage. Furthermore, we investigated the role dicarbonyl stress may have on this increased susceptibility to DNA damage under folic acid-deficient conditions. Herein we reveal a significant interaction between folic acid and glucose or MGO with respect to MNi frequency. Furthermore, we show that aminoguanidine, which can scavenge both MGO and free radicals, was able to prevent glucose-induced DNA damage in folic acid-deficient cells. Our results suggest that glucose-induced dicarbonyl and oxidative stress exacerbates DNA damage under folic acid-deficient conditions.

## Methods

### Materials

All reagents, chemicals, and enzymes were purchased from Sigma unless indicated otherwise. All reagents for cell culture were of cell culture grade. Isotopically labeled and unlabeled MG-H1 (Nδ-(5-hydro-5-methyl-4-imidazolon2-yl)ornithine), CEL, and lysine were purchased from Iris biotech (Marktredwitz, Germany).

### Media preparation

High glucose RPMI (45 mmol/l) was prepared by dissolving 6.1 g of glucose (G7021, Sigma) in 1 l of standard RPMI (R0883, Sigma; LG, 11.1 mmol/l glucose). Media was then passed through a 0.2 µm filter under vacuum directly into a sterile container and stored at 4°C.

For the preparation of media with different concentrations of folic acid, RPMI-1640, 10× without folic acid (R1145, Sigma), was diluted 1:10 with 900 ml of preautoclaved Milli-Q. Sodium bicarbonate (2.0 g) was added, and the solution passed through a 0.2-µm filter under vacuum directly into a sterile container. This was repeated with the inclusion of 6.1 g of glucose for high glucose folic acid-free RPMI. For the preparation of 23 (deficient) and 226 (medium) nmol/l folic acid RPMI, standard RPMI (Replete; 2264 nmol/l) was diluted 1:100 and 1:10, respectively, with either low or high glucose folic acid-free RPMI to achieve the required final concentration of each ingredient.

### WIL2-NS cell culture

**Figure 1.**
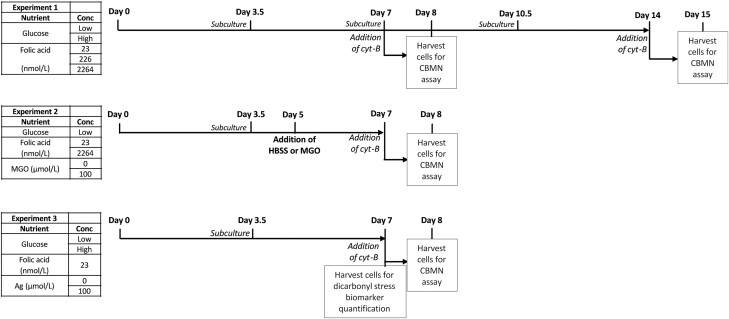
Culturing procedure for experiments 1–3. Experiment 1: WIL2-NS were cultured in LG or HG with varying concentrations of folic acid for 14 days. Cells were subcultured every 3.5 days and CBMNcyt assay performed on days 7 and 14. Experiment 2: WIL2-NS were cultured under folic acid-deficient or replete conditions for 7 days and subcultured after 3.5 days. MGO or vehicle (HBSS) was added to the media on day 5 for a further 48 h and CBMNcyt assay was performed on day 7. Experiment 3: cells were cultured under folic acid deficient conditions in either LG or HG media for 7 days. Cells were also cultured in the presence or absence of aminoguanidine (100 µmol/l). Cells were subcultured on day 3.5 and CBMNcyt assay was performed on day 7. Ag, aminoguanidine; HG, high glucose (45 mmol/l glucose); LG, low glucose (11.1 mmol/l glucose).

The WIL2-NS (ATCC CRL-8155) cell line was kindly gifted by the Commonwealth Scientific Research Organization (Adelaide, Australia). Cells were grown in RPMI supplemented with 5% (v/v) fetal calf serum, l-glutamine (1%, v/v), and penicillin/streptomycin (1%, v/v) at 37°C in a humidified atmosphere with 5% CO_2_. This study involved three experiments, which differed based on the medium composition of folic acid, glucose, or MGO as well as culturing duration and are described in Fig. 1. Briefly, in experiment 1, cells were seeded at 3 × 10^5^ cells/ml and subcultured back to the original seeding density every 3.5 days. On days 7 and 14, an aliquot of cells (1.5 × 10^5^ cells) was used for the CBMNcyt assay in which cytochalasin-B (cyt-B; 4.5 µg/ml) was added and cells harvested 24 h later and prepared as described below. For experiment 2, cells were seeded at 3 × 10^5^ cells/ml and subcultured back to original seeding density on day 3.5 and treated with vehicle (Hanks balanced salt solution, HBSS) or MGO (100 µmol/l) on day 5. The CBMNcyt assay was set up on day 7 by the addition of cyt-B (4.5 µg/ml) and harvested 24 h later. For experiment 3, the folic acid concentration was kept constant at 23 nmol/l across all samples, and glucose concentrations were either 11.1 (low) or 45 mmol/l (high). Cells were either cultured in the presence of aminoguanidine (MGO scavenger; 100 µmol/l) or vehicle (HBSS). Cells were seeded at 3 × 10^5^ and subcultured back to original seeding density every 3.5 days. On day 7, an aliquot of cells (1.5 × 10^5^ cells) was removed for the CBMNcyt assay (as described for experiments 1 and 2) and remainder were harvested for quantification of dicarbonyl stress biomarkers.

### CBMNcyt assay

The CBMNcyt assay is a comprehensive system for measuring DNA damage, cytostasis, and cytotoxicity. DNA damage events are scored specifically in once-divided binucleated cells and include (i) MNi, a biomarker of chromosome breakage and/or whole chromosome loss, (ii) NPBs, a biomarker of DNA misrepair and/or telomere end-fusions, and (iii) nuclear buds (NBUDs), a biomarker of elimination of amplified DNA and/or unresolved DNA repair complexes. Cytostatic effects are measured via the proportion of mono-, bi-, and multinucleated cells to calculate the nuclear division index (NDI), and cytotoxicity is determined via necrotic and/or apoptotic cell ratios. These biomarkers are identified microscopically using well-established and validated criteria as previously described [23]. At days 7 and/or 14, an aliquot of cells was removed and subcultured to 3 × 10^5^ cells/ml (in 0.5 ml of media) and treated with cytochalasin-B (cyt-B; 4.5 µg/ml) [23]. Cells were harvested 24 h later, and slide preparation and scoring of the CBMNcyt assay were performed as described previously [23, 24] using a NanoZoomer S60 (Hammatsu Photonics).

### Quantification of MGO in cells and media

MGO was quantified by derivatization with *o*-phenylenediamine (O-PD) to 2-methylquinoxaline (2-MQ) as previously described with some modification [25, 26]. Briefly, 20 µl of 20% trichloroacetic acid (w/v)/0.9% saline (w/v) was added to 40 µl of media or cell pellet (~2 × 10^6^ cells resuspended in 40 µl of ddH_2_O). Samples were briefly vortexed, and 40 µl of ddH_2_O was added to the sample. Cell samples were sonicated for 30 s at 35% intensity (Misonix, NY, USA). Samples were then placed on a rotating incubator (150 rpm) for 10 min before being centrifuged at 14 000 *g* for 10 min. An aliquot (70 µl) was removed and mixed with 10 µl of 3% (w/v) sodium azide and 20 µl of 500 µM O-PD (in 200 mM HCl/500 µM diethylenetriaminepentaacetic acid). Samples were derivatized for 4 h in the dark. Following derivatization, 5 µl of presynthesized d_4_-2-MQ was added (prepared as above by reacting MGO with d_4_-O-PD (Toronto Research Chemicals, P319842)). Ten microliters of the derivatized solution were chromatographed with a 100 × 3 mm, 3 µm Gemini C18 (Phenomenex, Torrance, CA, USA) column with a linear gradient of 0.1% formic in water (Buffer A) and 0.1% formic acid in acetonitrile (Buffer B) over 9 min at a flow rate of 0.5 ml/min. Multiple reaction monitoring (MRM) was conducted in positive mode using an AB Sciex 6500+ QTRAP mass spectrometer with the following transitions: *m*/*z* 145.1 > 77.1 (2-MQ); *m*/*z* 149.1 > 77.1 (d_4_-2-MQ).

### Quantification of protein-bound MG-H1, CEL, and lysine

Measurement of AGEs from total cellular protein was performed as previously described with some modifications [27]. Briefly, cells (~5 × 10^6^) were collected and washed twice with phosphate-buffered saline. Cells were lysed by sonication at 35% for 90 s and the supernatant collected following centrifugation (17 000 *g* for 10 min). A total of 100 µg of protein was precipitated with ice-cold acetonitrile:methanol (3:1) and stored for 20 min at −20°C and then collected by centrifugation (17 000 *g* for 10 min). Supernatant was discarded and residual acetonitrile was removed under a stream of nitrogen. Samples were reconstituted in 20 µl of water. Following this, 10 µl of 100 mM HCl, 5 µl of pepsin (2 mg/ml in 20 mM HCl), and 5 µl of thymol solution (1 mg/ml in 20 mM HCl) were added. Samples were incubated at 37°C for 24 h on a shaking incubator (150 rpm). Following incubation, 12.5 µl of 100 mM potassium phosphate buffer, pH 7.4, was added to the sample. After this, 5 µl of 260 mM KOH, 5 µl of Pronase E (2 mg/ml in 10 mM potassium phosphate buffer, pH 7.4) and 5 µl of Pen-Strep (1000 units/ml and 1 mg/ml, respectively) were added to the mixture and samples were further incubated at 37°C for 24 h in a rotating incubator (150 rpm). Following this, 5 µl of aminopeptidase (2 mg/ml in 10 mM potassium phosphate buffer, pH 7.4) and 5 µl of prolidase (2 mg/ml in 10 mM potassium phosphate buffer, pH 7.4) was added, and samples were further incubated at 37°C for 48 h in a rotating incubator. Sample (1 µl) was injected and analytes were separated using a 150 × 4.6 mm, 4 µm Phenomenex C18 column (Phenomenex, Torrance, CA, USA) with a linear gradient of 0.1% formic in water (Buffer A) and 0.1% formic acid in acetonitrile (Buffer B) over 5 min at a flow rate of 0.6 ml/min. MRM was conducted in positive mode using an AB Sciex 6500+ QTRAP mass spectrometer with the following transitions *m*/*z* 147.4 > 83.9 (lysine), 151.2 > 87.9 (d_4_ lysine), 219.2 > 130.2 (CEL), 222.2 > 134.2 (d_4_ CEL), 229.2 > 116.1 (MG-H1), and 232.2 > 116.1 (d_3_ MG-H1).

### Quantification of free MG-H1 and CEL

Due to the low concentration of MG-H1 and CEL in cell culture media, a derivatization step was used to increase the sensitivity of their detection as previously described [28]. An aliquot of internal standard (100 nmol/l) was added to cell culture media (100 µl), which was deproteinized with acetonitrile: methanol (3:1). The supernatant was collected following centrifugation (17,000 *g*, 10 min) and dried under a stream of nitrogen. The sample was reconstituted in 1-butanol:HCl (3:1) and heated at 70°C for 90 min. The samples were then dried under nitrogen and reconstituted in 5% (v/v) MeOH. Standards were prepared identically to samples. Sample (5 µl) was injected and analytes were separated using a 150 × 4.6 mm, 4 µm Phenomenex C18 column (Phenomenex, Torrance, CA, USA) with 0.1% (v/v) formic in water (Buffer A) and 0.1% (v/v) formic acid in acetonitrile (Buffer B) over 10 min at a flow rate of 0.7 ml/min. MRM was conducted in positive mode using an AB Sciex 6500+ QTRAP mass spectrometer with the following transitions *m*/*z* 331.1 > 186.1 (CEL), 335.2 > 190.1 (d_4_ CEL), 285.1 > 172.1 (MG-H1), and 288.0 > 172.1 (d_3_ MG-H1).

### Quantification of media 8-oxo-2ʹ-deoxyguanosine

An aliquot of media was passed through a micro spin ultrafiltration (Amicon-Ultra; 10 kDa cutoff) column. Internal standard ([15N, 13C_2_] 8-oxo-2ʹ-deoxyguanosine [8-oxodG]) was added to a final concentration of 10 nmol/l. Sample (10 µl) was injected and the analyte was separated using a 100 × 3 mm, 3 µm Gemini C18 (Phenomenex, Torrance, CA, USA) column with 0.1% (v/v) formic in water (Buffer A) and 0.1% (v/v) formic acid in acetonitrile (Buffer B) over 10 min at a flow rate of 0.5 ml/min. An aliquot of [15N_5_, 13C_10_] 2-deoxyguanosine (2-dG) was also added (10 nmol/l) to ensure both analytes were resolved to prevent overestimation of 8-oxodG by in source formation from oxidation of 2-dG. MRM was conducted in positive mode using an AB SCIEX 6500+ QTRAP with the following transitions: *m*/*z* 284.2 > 168.0 (8-oxodG) and 287.2 > 171.0 ([15N, 13C_2_] 8-oxodG).

### Statistical analysis

Data analysis was performed using GraphPad Prism (San Diego, CA, USA, Version 8.0.0). For experiments 1 and 2, a two-way analysis of variance (ANOVA) followed by Bonferroni *post hoc* test was conducted to compare the statistical significance of differences in the effect of glucose or MGO at each concentration of folic acid and to determine the percentage of variance explained by each variable and their interaction. One-way ANOVA followed by Dunnett *post hoc* test was conducted when comparing between groups of experiment 3. A *P* < .05 was considered significant, and the results were expressed as mean ± SD from three to four independent experiments.

## Results

### Folic acid-deficient cells are sensitive to glucose-induced DNA damage

We hypothesized that the interaction between folic acid and glucose was a result of elevated dicarbonyl stress. To address this, we first evaluated the intracellular MGO concentrations of WIL2-NS cells cultured under high glucose (45 mmol/l) versus low glucose (11.1 mmol/l) conditions. Cells cultured in high glucose for 7 days showed a 6-fold increase in the concentration of intracellular MGO compared with those cultured under low glucose, thus confirming it as an appropriate model of dicarbonyl stress (Supplementary Fig. 1). We next evaluated the interaction between folic acid and glucose with respect to cell viability, proliferation and DNA damage biomarkers as measured by the CBMNcyt assay (NDI, necrosis, MNi, NPB, and NBUD). Folic acid deficiency caused a dose-dependent reduction in proliferation and viability over a 14-day period, though, at 2264 and 226 nmol/l, the addition of high glucose had no impact (Supplementary Fig. 2A and B). However, at 23 nmol/l folic acid, high glucose resulted in a further significant reduction in proliferation and viability on day 14 (Supplementary Fig. 2A and B). To confirm this effect of high glucose in a folic acid-deficient environment was not caused by osmotic stress, the additional glucose in these cultures was replaced with nonmetabolizable mannitol. No significant difference was observed between high mannitol and low glucose at any concentration of folic acid for proliferation and viability (Supplementary Fig. 2C and D).

**Figure 2.**
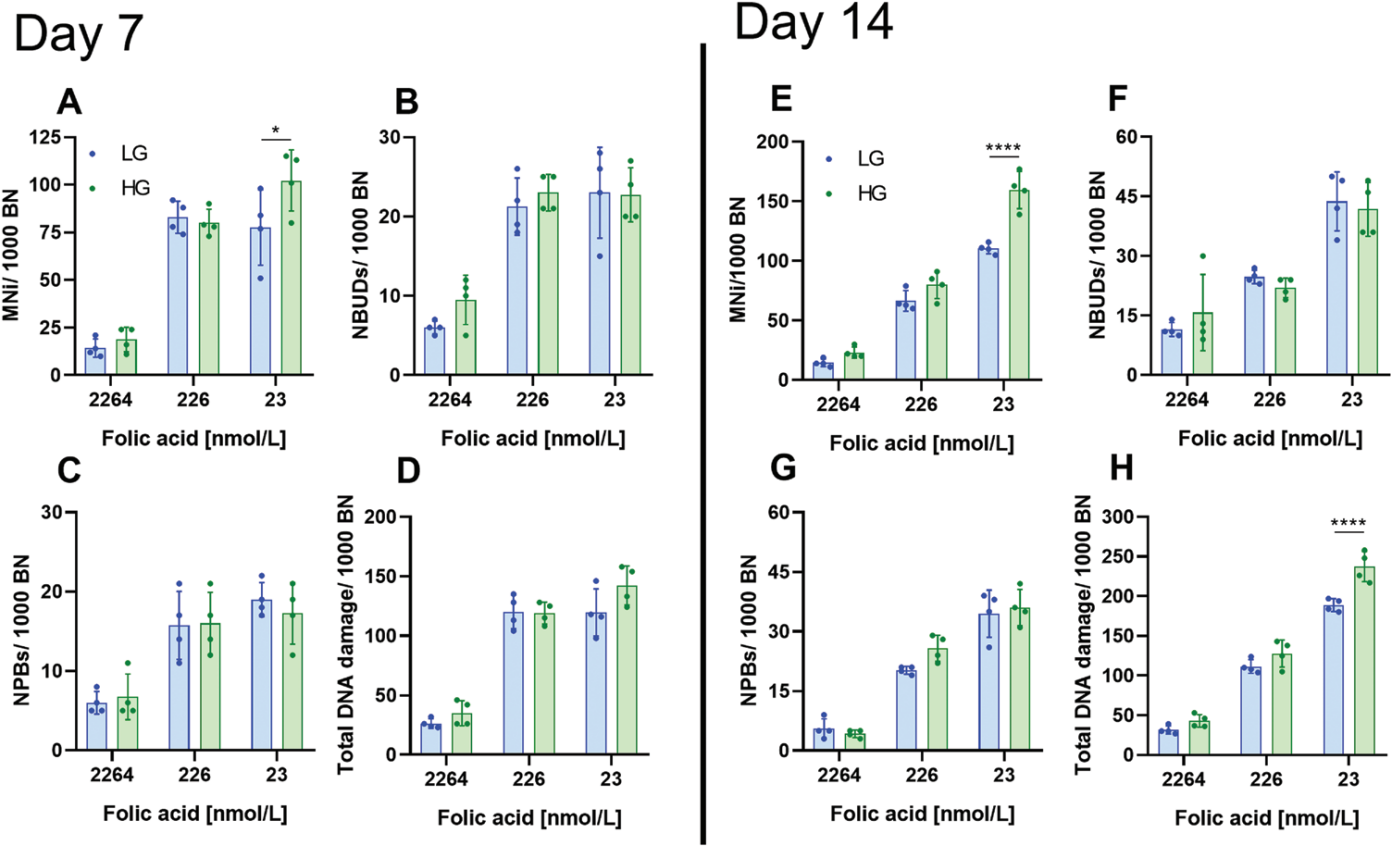
Folic acid-deficient WIL2-NS cells are sensitive to glucose-induced DNA damage. (A–D) are DNA damage biomarkers measured at day 7, (E, F) are DNA damage biomarkers measured at day 14. (A, E) MNi/1000 BN. (B, F) NBUDs/1000 BN. (C, G) NPBs/1000 BN. (D, H) Total DNA damage/1000 BN. HG, high glucose; LG, low glucose. Values represent mean ± SD of *n* = 4. ∗*P* < .05, ∗∗∗∗*P* < .0001.

We next measured cytogenetic and DNA damage biomarkers on days 7 and 14. No significant difference was observed between low and high glucose cultures at any concentration of folic acid for NDI, frequency of necrotic cells, NBUDs, or NPBs at day 7 or 14 (Supplementary Fig. 3A–D, Fig. 2B–D, F, and G). However, at days 7 and 14, high glucose increased the frequency of MNi in cells cultured in 23 nmol/l folic acid, but not in 226 or 2264 nmol/l (Fig. 2A and E). Furthermore, high glucose increased total DNA damage in folic acid-deficient cells on day 14 (Fig. 2H). To confirm the observed increase in MNi frequency and total DNA damage was not a result of osmotic stress, high mannitol cultures were used. No significant difference was observed for MNi frequency or total DNA damage between low glucose and high mannitol under folic acid-deficient conditions at day 7 or 14 (Supplementary Fig. 4A–C). This supports our hypothesis that the effect of glucose in folic acid-deficient cells is a downstream effect of glucose metabolism. A two-way ANOVA was conducted to determine if there was a significant interaction between folic acid and glucose concentration on cytogenetic and DNA damage biomarkers. No significant interaction was observed at day 7 for any biomarker. However, on day 14, a significant interaction was observed for MNi frequency and total DNA damage (Table 1). These results corroborate previous findings demonstrating folic acid-deficient cells are more sensitive to glucose-induced DNA damage than those under replete conditions [17].

**Table 1.** Two-way ANOVA of CBMNcyt assay biomarkers to determine effect of folic acid, glucose, and their interaction.

		Source of variation					
		Day 7			Day 14		
		Folic acid	Glucose	Interaction	Folic acid	Glucose	Interaction
NDI	Percentage (%)	5.46	0.19	9.53	15.09	2.95	5.35
	*P* value	.19	.84	.38	.1983	.4159	.5450
Necrosis (%)	Percentage (%)	**79.63**	0.059	0.65	**46.49**	**10.83**	5.69
	*P* value	**<.0001**	.82	.74	**.0007**	**.0339**	.2761
MNi	Percentage (%)	**87.22**	1.55	2.79	**88.79**	**5.51**	**3.20**
	*P* value	**<.0001**	.0855	.0768	**<.0001**	**<.0001**	**.0006**
NBUDs	Percentage (%)	**82.35**	1.180	01.00	**83.89**	0.00	1.409
	*P* value	**<.0001**	.2566	.5700	**<.0001**	.9541	.4385
NPBs	Percentage (%)	**75.90**	0.046	0.8537	**92.74**	0.55	1.14
	*P* value	**<.0001**	.8527	.7224	**<.0001**	.2001	.1860
Total DNA damage	Percentage (%)	**91.63**	1.179	1.084	**93.83**	**2.94**	**1.23**
	*P* value	**<.0001**	.0787	.2299	**<.0001**	**<.0001**	**.0135**

Source of variation was measured by two-way ANOVA and expressed as percentage with corresponding *P* value. Values in bold are considered a significant source of variation to that biomarker.

### Folic acid-deficient cells are sensitive to MGO

**Figure 3.**
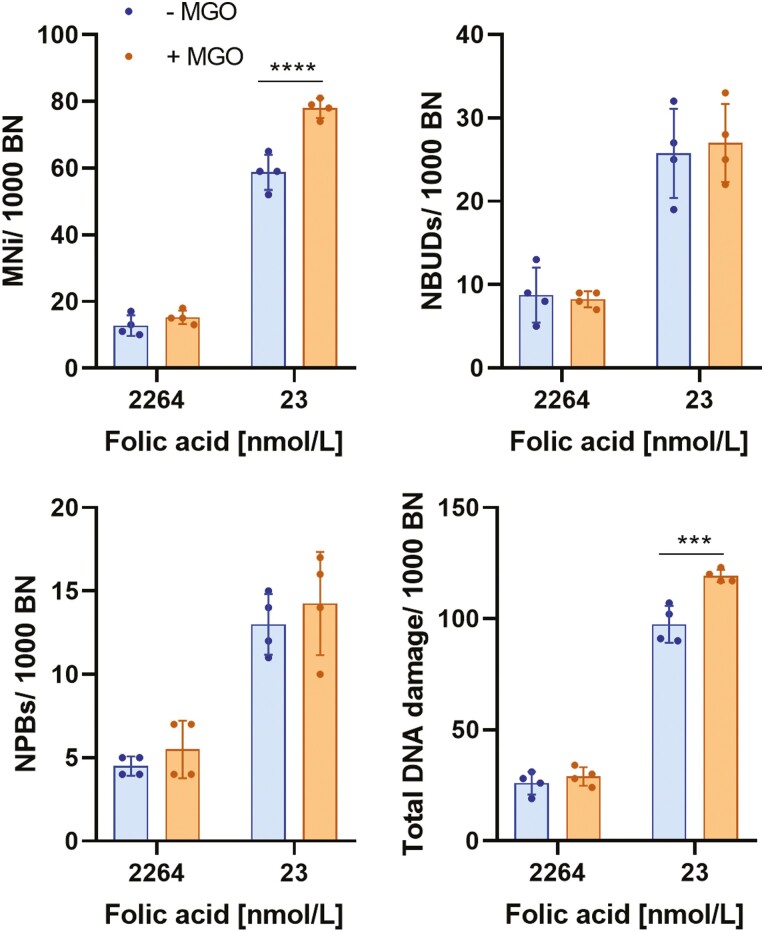
Folic acid-deficient WIL2-NS cells are sensitive to MGO-induced DNA damage. WIL2-NS cells were cultured under low (22.64 nmol/l) or high folic acid (2264 nmol/l) and treated with either HBSS (vehicle) or MGO (100 µmol/l) on day 5. (A) MNi/1000 BN. (B) NBUDs/1000 BN. (C) NPBs/1000 BN. (D) Total DNA damage/1000 BN. Values represent mean ± SD of *n* = 4. ∗∗∗*P* < .001, ∗∗∗∗*P* < .0001.

Elevated concentrations of glucose can increase intracellular levels of MGO [21, 29]. Therefore, we sought to determine if folic acid-deficient cells (23 nmol/l) are more sensitive to MGO than those under replete conditions (2264 nmol/l). To test this, cells were cultured for 7 days in either folic acid replete or deficient media and treated either with HBSS (vehicle) or MGO (100 µmol/l) on day 5 for 48 h. Under folic acid replete conditions, MGO had no effect on any of the DNA damage biomarkers measured (Fig. 3A–D). However, under folic acid-deficient conditions, the frequency of MNi and total DNA damage was significantly increased compared with untreated cells (Fig. 3A–D). Furthermore, two-way ANOVA analysis showed the interaction between folic acid and MGO was a significant source of variation for MNi and total DNA damage (Table 2).

**Table 2.** Two-way ANOVA of CBMNcyt assay biomarkers to determine effect of folic acid, MGO, and their interaction.

		Source of variation		
		Folic acid	MGO	Interaction
MNi	Percentage (%)	**92.89**	**3.72**	**2.20**
	*P* value	**<.0001**	**<.0001**	**.0005**
NBUDs	Percentage (%)	**86.95**	0.04	0.21
	*P* value	**<.0001**	.8530	.6665
NPBs	Percentage (%)	**84.67**	1.44	0.18
	*P* value	**<.0001**	<.2861	.9033
Total DNA damage	Percentage (%)	**95.17**	**2.23**	**1.28**
	*P* value	**<.0001**	**<.0007**	**.0052**

Source of variation was measured by two-way ANOVA and expressed as percentage with corresponding *P* value. Values in bold are considered a significant source of variation to that biomarker.

### Aminoguanidine rescues high glucose-induced DNA damage in folic acid-deficient cells

**Figure 4.**
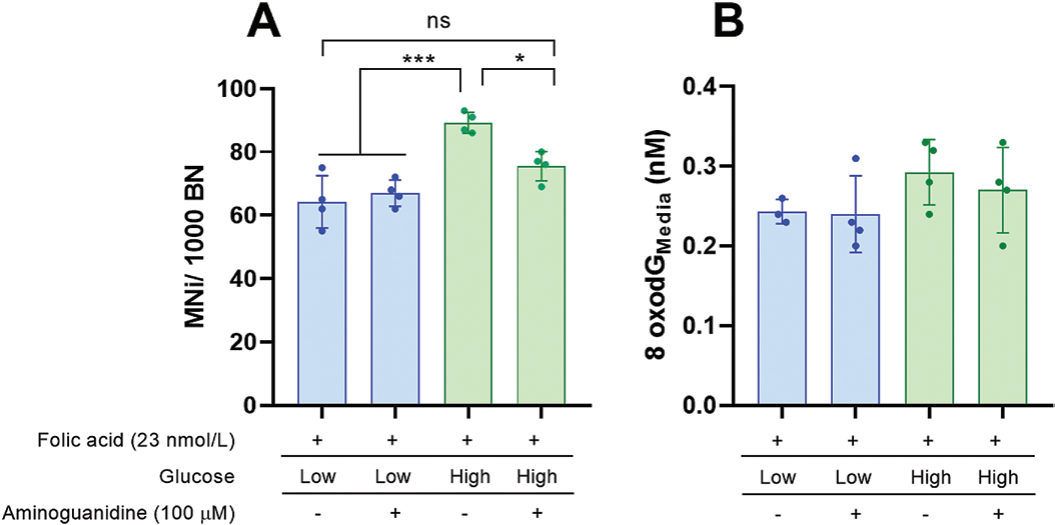
Aminoguanidine prevents glucose-induced DNA damage in folic acid-deficient cells. WIL2-NS cells were cultured in folic acid-deficient (23 nmol/l) RPMI under low or high glucose with or without aminoguanidine (100 µmol/l. (A) MNi/1000 BN. (B) Medium 8-oxodG (nM). Values represent mean ± SD of *n* = 4. ∗*P* < .05, ∗∗∗*P* < .001.

We next investigated if the sensitivity of folic acid-deficient cells to glucose-induced DNA damage was a result of elevated MGO. To test this, cells were cultured in low or high glucose under folic acid-deficient conditions (23 nmol/l) and exposed to either vehicle (HBSS) or aminoguanidine (100 µmol/l), a well-validated MGO scavenger [30]. Consistent with our previous results, high glucose increased the frequency of MNi in folic acid-deficient conditions (Fig. 4A). Interestingly, when exposed to aminoguanidine, high glucose no longer increased MNi frequency under folic acid-deficient conditions (Fig. 4A). To ensure the protective effect of aminoguanidine was specific to high glucose cultures, cells under low glucose and folic acid-deficient conditions were also exposed to 100 µmol/l aminoguanidine. Aminoguanidine did not reduce the frequency of MNi in cells cultured under folic acid-deficient and low glucose conditions. This supports our hypothesis that the interaction between glucose and folic acid was at least in part due to glucose-induced dicarbonyl stress.

To further explore the interaction between glucose-induced MNi under folic acid-deficient conditions and what role dicarbonyl stress may have had, we investigated various dicarbonyl stress biomarkers. High glucose significantly increased the concentration of cellular MGO by ~4-fold when compared with low glucose under folic acid-deficient conditions; however, this was not impacted by the presence of aminoguanidine (Table 3). Furthermore, no differences were observed for the media concentration of MGO, protein-bound and free concentrations of MG-H1 and CEL when high glucose cells were exposed to aminoguanidine (Table 3). Since glucose has previously been shown to induce oxidative DNA damage, we quantified the media concentration of 8oxodG as a surrogate measure of oxidative stress. No significant difference was observed between low and high glucose cultures regardless of treatment with aminoguanidine (Fig. 4B); however, 8-oxodG was significantly correlated with the frequency of MNi (*R*^2^ = 0.9301; *P* = .0356).

**Table 3.** Dicarbonyl stress biomarkers measured in WIL2-NS cells cultured under folic acid-deficient conditions (23 nmol/l) in LG or HG, with or without aminoguanidine.

Biomarker	Sample			
	LG	LG + aminoguanidine	HG	HG + aminoguanidine
Media MGO (pmol/10^6^ cells)	39.70 ± 11.74	43.88 ± 5.67	46.04 ± 4.49	39.23 ± 11.27
Cellular MGO (relative abundance)	1.00 ± 0.26	1.22 ± 0.41	3.43 ± 1.15∗∗	3.95 ± 1.33∗∗
MG-H1 (mmol/mol lysine)	0.15 ± 0.05	0.13 ± 0.04	0.18 ± 0.04	0.12 ± 0.04
CEL (mmol/mol lysine)	0.06 ± 0.02	0.04 ± 0.02	0.05 ± 0.01	0.04 ± 0.01
MG-H1_media_ (pmol/10^6^ cells)	79.14 ± 8.11	65.02 ± 15.56	76.57 ± 23.51	61.27 ± 21.41
CEL_media_ (pmol/10^6^ cells)	1.60 ± 0.38	0.91 ± 0.33	1.08 ± 0.18	1.43 ± 0.60

Data are mean ± SD, *n* = 4. HG, high glucose; LG, low glucose.

∗∗Significance: *P* < .01 when compared with LG.

### Folic acid deficiency increases dicarbonyl stress biomarkers

**Figure 5.**
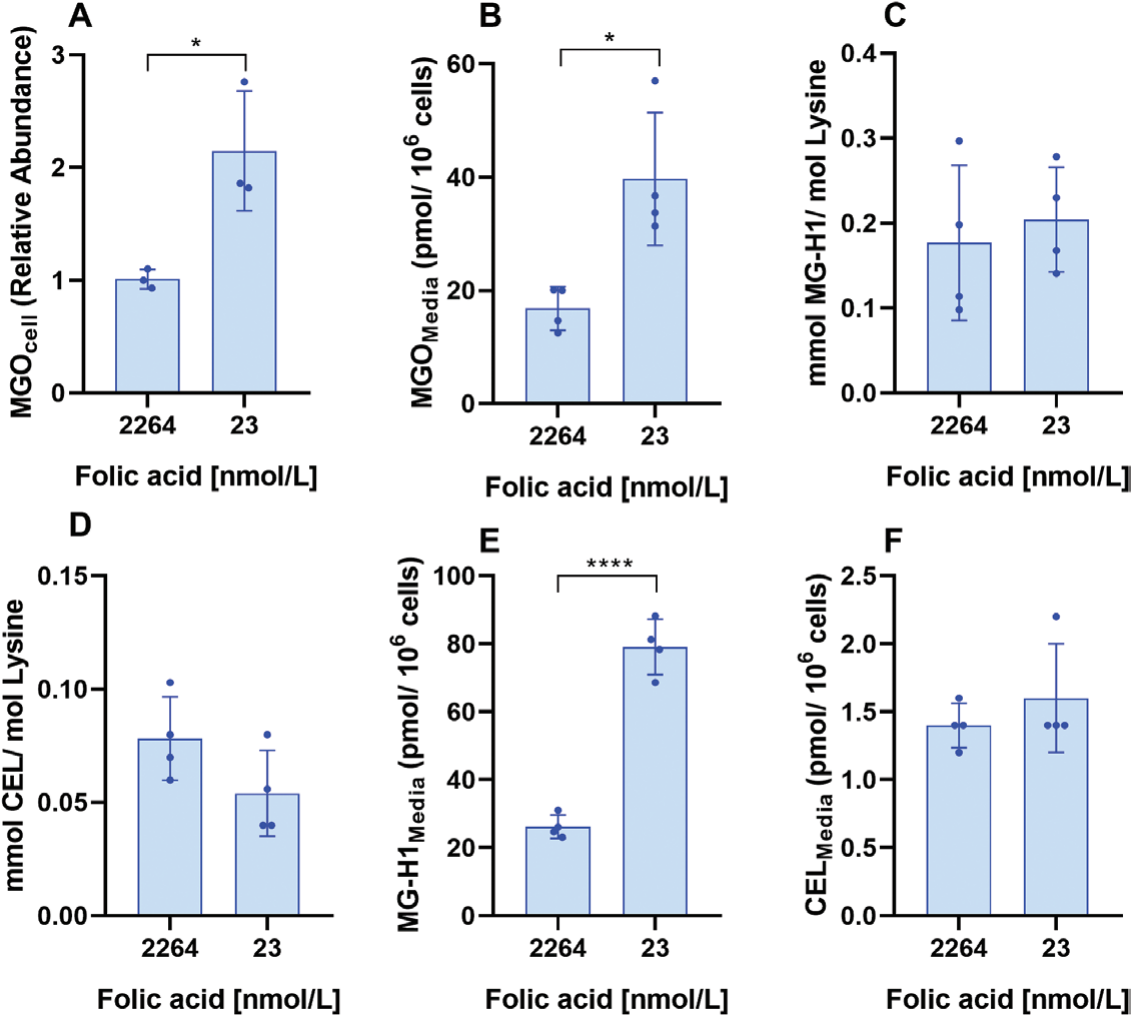
Folic acid deficiency increases concentration of dicarbonyl stress biomarkers. WIL2-NS cells were cultured in folic acid replete (2264 nmol/l) or deficient (23 nmol/l) RPMI under LG for 7 days. (A) Intracellular MGO expressed as relative abundance to control. (B) Medium MGO normalized to cell number (pmol/10^6^ cells). (C) Protein-bound concentration of MG-H1 normalized to lysine content (mmol/mol lysine). (D) Protein-bound concentration of CEL normalized to lysine content (mmol/mol lysine). (E) Medium concentration of MG-H1 normalized to cell number (pmol/10^6^ cells). (F) Medium concentration of CEL normalized to cell number (pmol/10^6^ cells). Values represent mean ± SD of *n* = 3–4. ∗*P* < .05, ∗∗∗∗*P* < .0001.

Interestingly, we observed from the previous section that under folic acid-deficient conditions, cells cultured under high glucose experience a smaller increase (3.4-fold; Table 3) in intracellular MGO than those cultured under folic acid replete conditions (6-fold; Supplementary Fig. 1). We hypothesized that this may be due to folic acid-deficient cells already having higher levels of intracellular MGO than those under replete conditions, which would account for the smaller increase in MGO following addition of high glucose. Therefore, we compared the concentration of various dicarbonyl stress biomarkers between WIL2-NS in folic acid replete and deficient media. We found a significant increase in both cellular (2.14-fold) and media (2.36-fold) concentrations of MGO in folic acid-deficient cells when compared with replete controls (Fig. 5A and B). We next investigated the concentration of protein-bound MG-H1 and CEL, however no difference was observed between folic acid replete and deficient controls (Fig. 5C and D). Lastly, we measured the media concentration of MG-H1 and CEL. From this, we found no significant difference in media CEL, however, media concentrations of MG-H1 significantly increased from 26.21 ± 3.40 pmol/10^6^ cells in folic acid replete controls to 79.14 ± 8.11 pmol/10^6^ cells in deficient cells (Fig. 5E and F). This suggests that folic acid-deficient cells may experience higher levels of dicarbonyl stress, irrespective of glucose concentration.

## Discussion

Accumulation of intracellular MGO and the formation of AGEs have been implicated in the pathogenesis of various diabetic complications. However, little is known about their role in CIN. We recently reported that MGO induces CIN and mitotic dysfunction in WIL2-NS and peripheral blood lymphocytes [15]. In this current study, we show that cells deficient in folic acid are more sensitive to glucose- or MGO-induced DNA damage. We also provide evidence that the increased sensitivity to glucose under folic acid-deficient conditions may be in part be due to elevated concentrations of cellular MGO. Furthermore, we reveal that deficiency in folic acid leads to elevated MGO and other dicarbonyl stress biomarkers. Understanding these interactions is important as various pathological states, such as T2D, are associated with altered metabolism and/or deficiencies in various micronutrients.

Data herein support previous findings that folic acid-deficient cells are more sensitive to glucose-induced DNA damage than those under replete conditions [17].

Under folic acid-deficient conditions, WIL2-NS cells showed an elevated propensity for MNi induction when exposed to glucose or MGO. The reactive glycolytic byproduct MGO, is a major metabolite produced under hyperglycemic conditions and can be elevated up to 8-fold in T2D [31]. Under *in vitro* conditions, high concentrations of glucose can increase intracellular MGO concentrations by up to 6-fold, which is consistent with the increase observed in this study [21, 32]. To determine if elevated MGO influenced the interaction between folic acid and glucose with respect to MNi frequency, the dicarbonyl scavenger aminoguanidine was used. Under folic acid conditions, the addition of aminoguanidine to high glucose cultures reduced the MNi frequency back to similar levels seen in the low glucose cultures. Furthermore, we showed that this protective effect of aminoguanidine was specific to high glucose cultures and showed no benefit under folic acid-deficient conditions when glucose levels were low. However, although aminoguanidine did reduce the frequency of MNi, it had no effect on intracellular MGO or other dicarbonyl stress biomarkers. Therefore, this suggests the protective effect of aminoguanidine is independent of its MGO scavenging activity. Aminoguanidine has previously been shown to inhibit inducible nitric oxide synthase and contain radical scavenging activity [33, 34]. High concentrations of glucose in cell culture model systems have been shown to increase the production of reactive oxygen species (ROS), which can cause oxidative DNA damage and subsequently MNi formation [35, 36]. In this study, we showed that MNi frequency was significantly correlated with the medium concentration of 8-oxodG. Therefore, high glucose may have stimulated ROS production, further increasing DNA damage and MNi formation, which was then rescued by radical scavenging activity of aminoguanidine. A significant interaction was also observed between folic acid and MGO. MGO has also been shown to stimulate production of ROS, which may have further influenced MNi formation [37]. Furthermore, we have shown MGO induces whole chromosome loss events in WIL2-NS cells which may have been perpetuated under folic acid conditions, which has been shown to impair spindle assembly checkpoints [15, 38].

Interestingly, in the previous study by Guo *et al.* the authors also observed an interaction between folic acid and other sugars, fructose, galactose, and sucrose with respect to DNA damage biomarkers [17]. When studying glycation, glucose is usually the metabolite of interest due to its high intracellular concentrations, relative to other sugar. However, other reducing sugars, such as fructose and galactose can also react with lysine and arginine residues via the Maillard reaction forming AGEs [39, 40]. The concentration of these sugars in most tissues is significantly lower than glucose, however, in cells from kidney and lens, especially in diabetic patients where concentration of fructose can reach up to 15 mM, their effect may be more pronounced [41]. Furthermore, fructose is metabolized by the liver, and enters the glycolytic pathway where it may contribute to MGO formation [42]. Therefore, under folic acid conditions, other sugar metabolites may also be genotoxic due to their glycation potential which have contributed to the interaction with folic acid observed previously [17].

In this study, we found that cells deficient in folic acid had significantly higher levels of cellular MGO among other dicarbonyl stress biomarkers. Interestingly, it has previously been shown that pyridoxal-phosphate (active form of vitamin B6), which is also critical in one-carbon metabolism deficiency increases the concentration of AGEs in HeLa and *D. melanogaster*, and this was associated with increased CIN [18]. Furthermore, pyridoxal-phosphate deficiency was associated with elevated intracellular glucose concentrations, however, as this vitamin is a cofactor for many enzymes, it is unclear if its role in one-carbon metabolism was involved [18, 19]. Although MGO is primarily formed as a byproduct of glycolysis, other pathways such as threonine and glycine interconversion can also contribute under specific circumstances [43–45]. Origin of MGO can be discerned by stable isotope experiments with [U-^13^C_6_] glucose and [U-^13^C_2_] glycine where the former produces fully labeled MGO (mass + 3 Da) and the latter produces MGO with one labeled carbon (mass + 1 Da) [46]. Nevertheless, it seems unlikely the elevated concentration of endogenous MGO significantly contributed to the observed DNA damage in folic acid-deficient cells, as addition of aminoguanidine showed no protective benefit. However, the large increase in DNA damage (4.6-fold) induced by folic acid deficiency may also have overshadowed any impact by MGO. Nevertheless, persistent dicarbonyl stress, even at low levels may have significant pathophysiological impacts.

Numerous studies have observed elevated frequency of MNi in T2D [7–11]. MNi frequency has been shown to be a prospective biomarker for cancer incidence with a 1.84- and 1.53-fold increase in relative risk for individuals with medium and high MNi frequency, respectively [47]. This is important considering the observations that individuals with T2D have an increased cancer risk [48]. Therefore, based on the results observed in this study, folic acid deficiency in T2D may potentiate the genotoxic effects of elevated glucose concentrations which may be associated with increased cancer risk observed in these individuals. In support of this, several studies have shown a decrease in MNi frequency of T2D following folic acid supplementation [49, 50]. However, whether this has any effect on cancer incidence has not been previously reported.

Overall, we found that folic acid-deficient WIL2-NS cells are sensitive to high concentrations of glucose, or the glycolytic byproduct MGO with respect to MNi formation. Furthermore, we showed that this interaction may be exacerbated by the production of ROS, which may be dependent on MGO formation. The present study provides further insight into the genotoxic interaction between folic acid and glucose, which is of significant importance due to the association between folate deficiency and T2D [13, 14].

## Supplementary Material

geac003_suppl_Supplementary_Material

## Data Availability

Study data will be made available by the corresponding author on reasonable request.
