# Supplementary material for: Folic acid deficiency increases sensitivity to DNA damage by glucose and methylglyoxal
Source: Mutagenesis. 2022 Jan 25;37(1):24–33. doi: 10.1093/mutage/geac003 (PMC9186029; doi:10.1093/mutage/geac003)
Supplement: geac003_suppl_Supplementary_Material [file geac003_suppl_supplementary_material.docx]

Supplementary Material

**Supplementary Figure 1. Intracellular MGO concentration under different glucose conditions.** LG; low glucose. HG; high glucose. Values represent mean ± SD of n=3. **p<0.01.

**Supplementary Figure 2. Proliferation and viability of WIL2-NS over 14-day period.** WIL2-NS cells were cultured in RPMI containing varying concentrations of folic acid, glucose or mannitol. **A)** No of viable cells. **B)** Cell viability determined by trypan blue. **C)** No of viable cells. **D)** Cell viability determined by trypan blue. No of viable cells in each condition was calculated by counting the numbers of viable cells in the culture at the time of harvesting and extrapolating to the numbers that would have been expected if all cells had been re-seeded at each split. LG; low glucose (11.1 mmol/L glucose). HG; high glucose (45 mmol/L glucose). HF; high folic acid (2264 nmol/L). MF; medium folic acid (226.4 nmol/L). LF; low folic acid (22.64 nmol/L). HM; high mannitol (33.9 mmol/L mannitol + 11.1 mmol/L glucose)**.** Values represent mean ± SD of n=4. **p<0.01, ***p<0.001.

**Supplementary Figure 3. Cytostatic and cytotoxic effects of glucose at different concentrations of folic acid. A)** Nuclear division index (NDI) at day 7. **B)** Frequency of necrotic cells at day 7. **C)** NDI at day 14. **D)** Frequency of necrotic cells at day 14. LG; low glucose. HG; High glucose. Values represent mean ± SD of n=4.

**Supplementary Figure 4. DNA damage in osmotic control WIL2-NS cultures.** Cells were cultured identically to those in experiment 1 (figure 1 & supplementary figure 2) except that the additional glucose in the high glucose cultures was replaced with non-metabolizable mannitol (33.9 mmol/L) **A)** MNi/ 1000 BN at day 7. **B)** MNi/ 1000 BN at day 14. **C)** Total DNA damage/ 1000 BN at day 14. LG; low glucose. HM; high mannitol. Values represent mean ± SD of n=3-4.
